# A multicenter real-world analysis of first-line systemic monotherapy for locally advanced basal cell carcinoma

**DOI:** 10.1016/j.jdcr.2023.10.006

**Published:** 2023-10-29

**Authors:** Morgan K. Groover, Neha Gupta, Emily Granger, Fadi Murad, Vernon J. Forrester, Emily J. Anstadt, William Su, Lauren Heusinkveld, John N. Lukens, Ann W. Silk, Allison T. Vidimos, Jonathan D. Schoenfeld, Shlomo A. Koyfman, Emily S. Ruiz

**Affiliations:** a Department of Dermatology, Brigham and Women’s Hospital, Harvard Medical School, Boston, Massachusetts; b Department of Dermatology, Cleveland Clinic, Cleveland Clinic Lerner College of Medicine, Cleveland, Ohio; c Department of Radiation Oncology, Perelman Center for Advanced Medicine, Perelman School of Medicine at the University of Pennsylvania, Philadelphia, Pennsylvania; d Department of Medical Oncology, Dana-Farber Cancer Institute, Boston, Massachusetts; e Department of Radiation Oncology, Brigham and Women’s Hospital, Harvard Medical School, Boston, Massachusetts; f Department of Radiation Oncology, Cleveland Clinic, Cleveland Clinic Lerner College of Medicine, Cleveland, Ohio

**Keywords:** basal cell carcinoma, competing-risk analysis, hedgehog inhibitors, immunotherapy, systemic therapy, treatment outcomes

National Comprehensive Cancer Network guidelines recommend systemic therapy (ST) for basal cell carcinoma when surgery or radiotherapy is unlikely to be curative or if a patient is a poor treatment candidate.^1^ The United States Food and Drug Administration-approved systemic therapies for locally advanced basal cell carcinoma (laBCC) are hedgehog inhibitors vismodegib and sonidegib, approved in 2012 and 2015, respectively; and programmed cell death protein-1 inhibitor cemiplimab, which was approved in 2021 for patients previously treated with an hedgehog inhibitor or in whom an hedgehog inhibitor is not appropriate.^2^, ^3^, ^4^ Since few patients require first-line ST, data on treatment and outcomes are limited. In this case series, our objective is to present real-world data on patient and tumor characteristics that lead to pursual of first-line ST for laBCCs and evaluate outcomes.

All patients who received care for laBCCs at Brigham and Women’s Hospital, Massachusetts General Hospital, Cleveland Clinic Foundation, or University of Pennsylvania Health System between January 1, 2005, and December 31, 2021, were identified. LaBCC was defined by histologically confirmed basal cell carcinoma cases that (1) required advanced surgical treatment, radiotherapy, or ST and (2) had not metastasized at time of presentation. Surgical criteria included advanced surgical resection (amputation, exenteration, or bone resection) or excision on the face or scalp of tumors >4 cm. The composite end point of poor outcomes included local recurrence, disease progression, metastasis, and death from disease.

A total of 594 laBCCs from 419 patients were identified, of which 20 tumors (3.4%) were treated first-line with ST alone (herein ST cohort) and 574 tumors (96.6%) were treated with any regimen including surgery, radiation, and/or ST (herein other therapy [OT] cohort). Tumors in the ST cohort were larger (ST: 55.7 mm [SD 36.4], OT: 33.2 mm [SD 34.1]; *P* = .01) and invaded deeper (ST: 9 beyond fat [45%]; OT: 140 beyond fat [24.4%]; *P* = .04), but incidence of aggressive histology was similar between cohorts (Table I). Most patients received first-line ST for an extensive or surgically unresectable tumor (15 patients, 75%) with the remainder having medical comorbidities that precluded other treatments (5 patients, 25%). In the OT and ST cohorts, 489 (85.2%) and 6 (30%) tumors achieved no evidence of disease, 55 (9.6%) and 12 (60%) tumors responded partially, and 16 (2.8%) and 1 (5%) tumor progressed, respectively (*P* < .0001). When adjusted for tumor diameter and depth, competing-risks regression showed 21.5% and 15.7% 5-year cumulative incidence of poor outcomes in the OT and ST cohorts, respectively (*P* = .85) (Fig 1). Median treatment duration in the ST cohort was 9.1 months (3.3-24.7). Notably, 9 patients (45%) stopped receiving ST due to side effects.

**Table I:** Patient and tumor characteristics of systemic therapy cohort and other therapy cohort of patients with locally advanced basal cell carcinoma

Patient characteristics	Other therapy (*N* = 399)	Systemic therapy (*N* = 20)
Male sex, *N* (%)	275 (68.9%)	11 (55.0%)
Age at diagnosis, mean (SD), y	70.0 (14.0)	77.2 (16.3)
Immunosuppressed, *N* (%)	45 (11.4%)	0 (0%)
Basal cell nevus syndrome, *N* (%)	3 (0.8%)	0 (0%)
Follow-up time, SD (months)	69.2 (65.4)	26.0 (16.0)

Tumor characteristics	Other therapy (*N* = 574)	Systemic therapy (*N* = 20)
Tumor diameter, mean (SD), mm	33.2 (34.1)	55.7 (36.4)
Tumor location, *N* (%)		
Head or neck	499 (86.9%)	17 (85.0%)
Other/unknown	75 (13.1%)	3 (15.0%)
Aggressive histology∗, *N* (%)	267 (46.5%)	12 (60.0%)
Perineural invasion	58 (10.8%)	2 (10.0%)
Tumor depth, *N* (%)		
Dermis/subcutaneous fat	434 (75.6%)	11 (55%)
Beyond subcutaneous fat	83 (14.5%)	7 (35%)
Bone	57 (9.9%)	2 (10%)
Reason for receiving systemic therapy as first-line treatment, *N* (%)		
Medical comorbidities	–	5 (25%)
Extensive/unresectable disease	–	15 (75%)
First-line systemic treatment, *N* (%)		
Vismodegib	–	17 (85%)
Sonidegib	–	1 (5%)
Pembrolizumab	–	2 (10%)
Poor outcome, *N* (%)		
Local recurrence	171 (29.8%)	2 (10%)
Metastasis	40 (7.0%)	0 (0%)
Death due to disease	18 (3.1%)	1 (5%)
Final response to treatment, *N* (%)		
No evidence of disease	489 (85.2%)	6 (30%)
Partial response	55 (9.6%)	12 (60%)
Disease progression	16 (2.8%)	1 (5%)
Unknown/lost to follow-up	14 (2.4%)	1 (5%)

∗ Micronodular, infiltrative, and squamous differentiation were considered aggressive, whereas nodular was considered nonaggressive.

**Fig 1:**
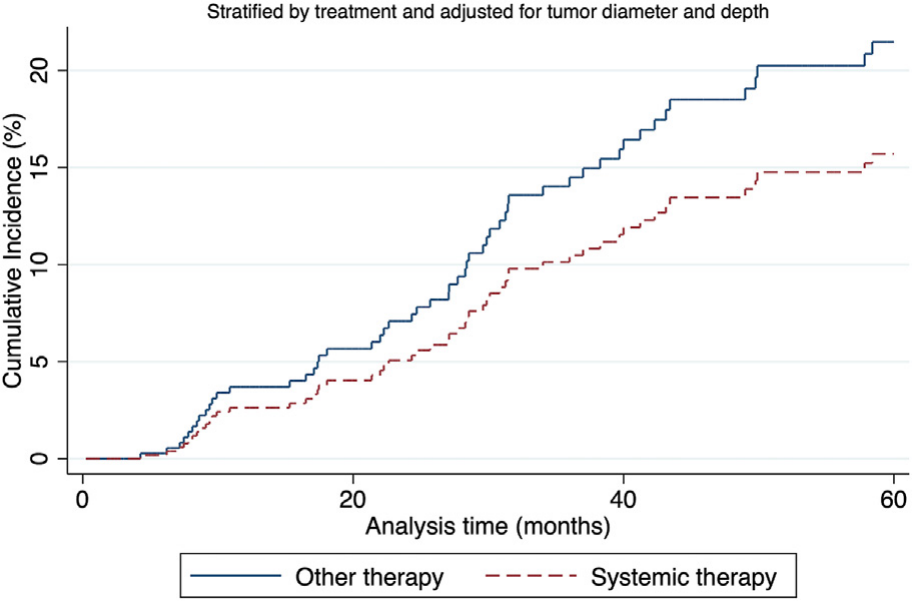
Competing-risk regression for poor outcomes in patients who received systemic therapy and other therapy for locally advanced basal cell carcinoma. Competing-risks regression, adjusted for tumor diameter and depth, shows 21.5% cumulative incidence of poor outcomes in the other therapy cohort and 15.7% 5-year cumulative incidence of poor outcomes in the systemic therapy cohort (*P* = .85). Poor outcome is a composite of local recurrence, disease progression, metastasis, and death from disease.

Although patients who received ST for advanced laBCC were less likely to achieve complete disease remission, ST allowed patients with significant disease and limited treatment options to live beyond 5 years without tumor progression, metastasis, or death from disease. Taken together, these real-world outcomes support that ST may be a viable option for patients with laBCC who are ineligible for other curative-intent treatment with surgery or radiotherapy.

## Conflicts of interest

Dr Ruiz received research support paid to the institution from Regeneron and PellePharm, and served as a consultant for Regeneron, Checkpoint Therapeutics, and Feldan Therapeutics. Dr Silk received research support paid to the institution from Biohaven Pharmaceuticals, Replimune, Morphogenesis, Shattuck Laboratories, Regeneron, and Merck, participated in the Regeneron Ad Board in July 2023, and served on the Board of Directors of the Society for Immunotherapy of Cancer. Dr Schoenfeld received research support paid to the institution from Merck, BMS, Regeneron, Debiopharm, and EMD Serono, served as a consultant or on the scientific advisory board or received travel fees from Castle Biosciences, Genentech, Immunitas, Debiopharm, BMS, Tilos, ACI Clinical, Astellas, Stimit, Merck KGA, SIRPant, and EMD Serono, received expert witness fees from Pearson Doyle Mohre & Pastis, participated in the data safety monitoring or advisory board of ACI Clinical, EMD Serano, and Merck KGA, had stock options in Immunitas, and had equity in Doximity. Dr Koyfman received research support from Merck, BMS, Castle Biosciences, and Regeneron, served as a consultant for Merck, Regeneron, and Galera, wrote for UpToDate, and participated in the advisory board for Alpha Part. Authors Groover and Ganger and Drs Gupta, Murad, Forrester, Anstadt, Su, Heusinkveld, Lukens, and Vidimos have no conflicts of interest to declare.
